# Evaluation of biochemical and reproductive biomarkers in pre and during gestational period in immunosuppressed Wistar rats with mycophelonate sodium

**DOI:** 10.1186/1758-5996-7-S1-A70

**Published:** 2015-11-11

**Authors:** Amanda Lima Deluque, Nagilla Orleanne Lima do Carmo, Elisangela Miranda de Jesus Lisboa, Gilsielle Benício Jaco, Betina Beatriz Mielke, Madileine Francely Américo, Luciana Aparecida Cora, Maria do Carmo Borges Teixeira, Kleber Eduardo de Campos

**Affiliations:** 1 Universidade Federal de Mato Grosso, Barra do Garças, Brazil

## Background

The pregnancy period involves a lot of biological changes, and the use of medication must be taken with extremely caution, especially because of its implications for maternal and fetal health, involving biochemical and reproductive functions in maternal organism. Also, the mycophenolate sodium (MFS) is used to avoid possible rejection of transplanted tissues.

## Objective

to evaluate the effects of maternal immunosuppression by MFS on reproductive and biochemical profile in rats pre and during pregnancy period.

## Materials and methods

The rats were divided into three groups: rats treated with water (CONT=10) rats orally treated with MFS (20mg/Kg) daily for 15 days until a positive diagnosis of pregnancy (MICO-1=10) and rats orally treated with MFS (20mg/Kg) daily for 15 days prior to and during 21 days of pregnancy (MICO-2=10). It was evaluated on the 17th day of pregnancy the Oral Tolerance Glucose Test (OGTT) and on day 21 the rats were anesthetized and killed by decapitation and thus measured reproductive parameters (Index fertility, childbirth, pregnancy and number of live births and resorption) and serum biomarkers (lipid parameters and liver transaminases) Statistical significance was p <0.05.

## Results

In pregnancy period, there were no changes in glucose data during OGTT evaluation, although MICO-2 rats presented higher glycemia compared to other groups (CONT and MICO-1), with 170mg/dL. Furthermore, the MICO-2 rats presented other negative effects, including dead rats and total resorption of fetuses. The biochemical data presented decreasing of rates of serum lipid (cholesterol and lipoprotein fractions, triglycerides and alanine aminotransferase).

## Conclusion

The continued use of MFS during pregnancy can be considered toxic, damaging the maternal and fetal health, lead to mild hyperglycemia, fetal loss with changes in maternal lipid metabolism. The association of immunossupression of MFS with gestational period is not recommended, and should be replaced by another immunosuppressant.

